# Revealing transient structures of nucleosomes as DNA unwinds

**DOI:** 10.1093/nar/gku562

**Published:** 2014-07-01

**Authors:** Yujie Chen, Joshua M. Tokuda, Traci Topping, Julie L. Sutton, Steve P. Meisburger, Suzette A. Pabit, Lisa M. Gloss, Lois Pollack

**Affiliations:** 1 School of Applied and Engineering Physics, Cornell University, Ithaca, NY 14853, USA; 2 School of Molecular Biosciences, Washington State University, Pullman, WA 99164, USA

## Abstract

The modulation of DNA accessibility by nucleosomes is a fundamental mechanism of gene regulation in eukaryotes. The nucleosome core particle (NCP) consists of 147 bp of DNA wrapped around a symmetric octamer of histone proteins. The dynamics of DNA packaging and unpackaging from the NCP affect all DNA-based chemistries, but depend on many factors, including DNA positioning sequence, histone variants and modifications. Although the structure of the intact NCP has been studied by crystallography at atomic resolution, little is known about the structures of the partially unwrapped, transient intermediates relevant to nucleosome dynamics in processes such as transcription, DNA replication and repair. We apply a new experimental approach combining contrast variation with time-resolved small angle X-ray scattering (TR-SAXS) to determine transient structures of protein and DNA constituents of NCPs during salt-induced disassembly. We measure the structures of unwrapping DNA and monitor protein dissociation from *Xenopus laevis* histones reconstituted with two model NCP positioning constructs: the Widom 601 sequence and the sea urchin 5S ribosomal gene. Both constructs reveal asymmetric release of DNA from disrupted histone cores, but display different patterns of protein dissociation. These kinetic intermediates may be biologically important substrates for gene regulation.

## INTRODUCTION

Packaging of DNA by proteins in the nucleosome core particle (NCP) affects all DNA-based chemistries, including transcription, replication, repair and recombination (1). The canonical NCP consists of 147 bp DNA wrapped around a symmetric histone octamer of two H2A–H2B heterodimers and an (H3–H4)_2_ tetramer. Because DNA accessibility is a prerequisite for initiating transcription and replication, an important unresolved question is how NCP disassembly proceeds to permit access to the DNA. Experiments carried out as a function of increasing salt (typically [NaCl] (2)) or force (3) reveal equilibrium intermediates with varying degrees of unwrapped DNA. Some contain bound, but disrupted protein cores (4–6). However, kinetic studies have been limited to Förster Resonance Energy Transfer (FRET) observations of spontaneous changes in DNA conformation (7–9) or to changes detected during protein binding (10–11). The former measurements reveal that the dissociation of the DNA ends from the NCP (often termed breathing) occurs on a time scale of 100–250 ms, while larger scale openings involving the release of internal DNA segments occur on the order of 1–10 min.

No kinetic experiments to date have monitored both the DNA and histone components of the NCP as DNA is released and the octamer core is disrupted. Here we describe a novel approach that combines contrast variation with time-resolved small angle X-ray scattering (TR-SAXS) to observe transient NCP structures following a rapid increase in salt. It is generally accepted that increasing concentrations of NaCl may populate intermediates species similar to those transiently populated at low ionic strength (5,12) and this perturbation approach facilitates the application of alternative biophysical methods to characterize intermediate species and relative time scales for their formation. Two unresolved questions that may have major functional roles for chromatin *in vivo* are addressed: (i) Is DNA release from the histone core symmetric or asymmetric? (ii) Do the eight histone proteins remain bound to DNA upon DNA unwrapping? Asymmetric disassembly of the nucleosome has been proposed based on the 5’-to-3’ processivity of DNA and RNA polymerases, and the ability of RNA pol II to displace H2A–H2B dimers (13). FRET-based models have generally assumed that the rapid DNA breathing motions are symmetrical (10), but transient NCP intermediates observed in recent high speed Atomic Force Microscopy (AFM) measurements detected asymmetrical opening on similar time scales (14). Equilibrium data indicate that the H2A–H2B dimers dissociate cooperatively from the NCP (6,15–17), but there is evidence for dissociation of one H2A–H2B dimer to form a hexasome kinetic intermediate (4,9,12,18).

We studied NCPs reconstituted with *Xenopus laevis* histones and two well-characterized 149 bp NCP-positioning sequences: the high affinity 601 DNA developed by the Widom lab (601-NCP) (6,19) and the weaker positioning ‘5S DNA’ from the promoter region of the *Lytechinus variegatus* (sea urchin) 5S ribosomal gene (5S-NCP) (20).

**Figure 1.**
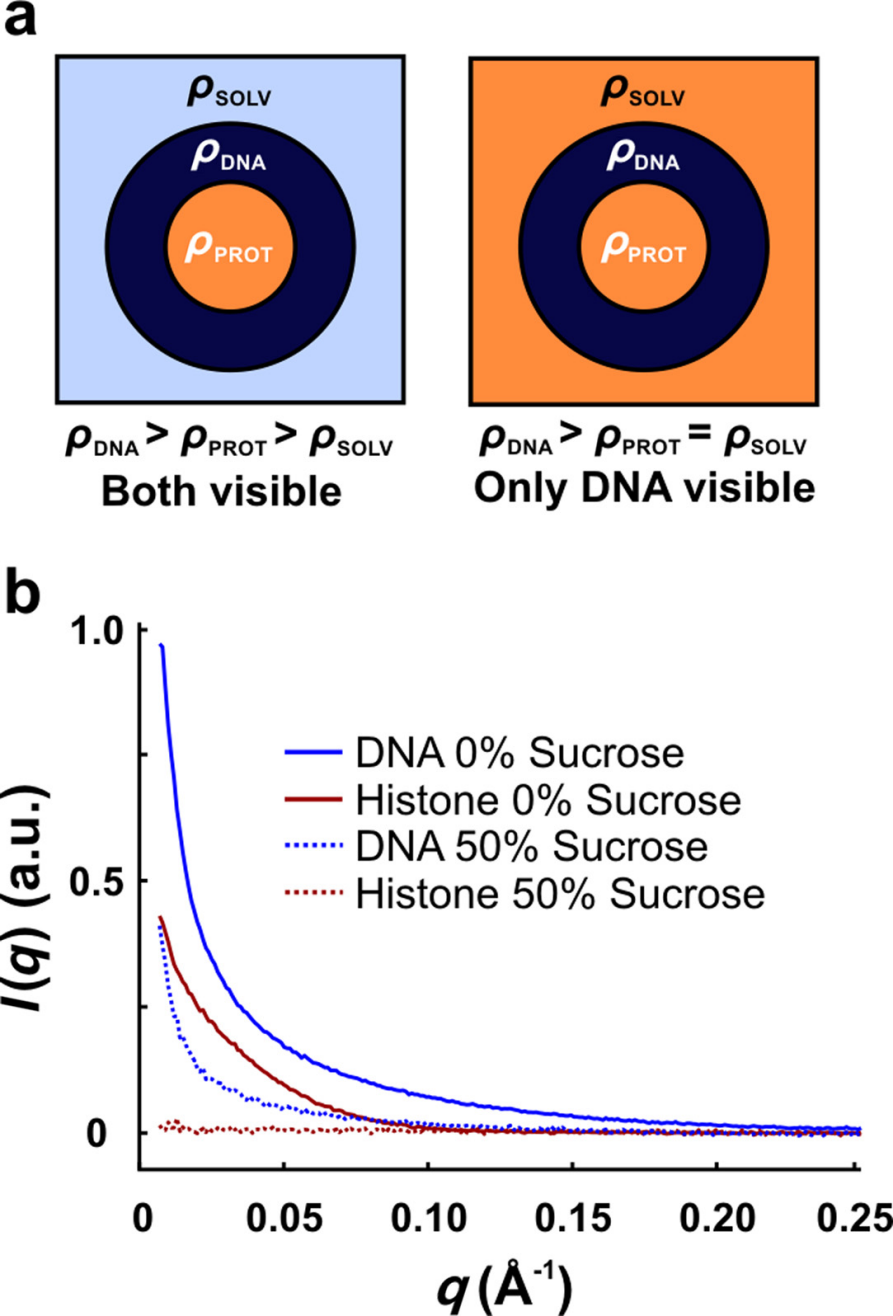
Contrast variation reveals DNA conformation within protein–nucleic acid complexes. (**a**) Cartoon depicting how contrast variation is used to isolate scattering from the DNA component in protein–nucleic acid complexes. Left*:* The protein–nucleic acid complex in solution can be approximated as three phases with electron densities *ρ*_SOLV_ (light blue), *ρ*_PROT_ (orange), and *ρ*_DNA_ (dark blue). Right*:* Because contrast arises from electron density differences, the electron density of the solvent is increased by adding small molecules such as sucrose until it matches that of the protein. Consequently, the protein is effectively ‘blanked’ and only the DNA contributes to the measured scattering signal. (**b**) Scattering profiles for NCP components measured separately in 2 M NaCl with and without sucrose. In 50% sucrose, proteins become invisible above the background and only the DNA contributes to the scattering. The resulting signal for the DNA is decreased because of the reduced contrast between the DNA and solvent.

**Figure 2.**
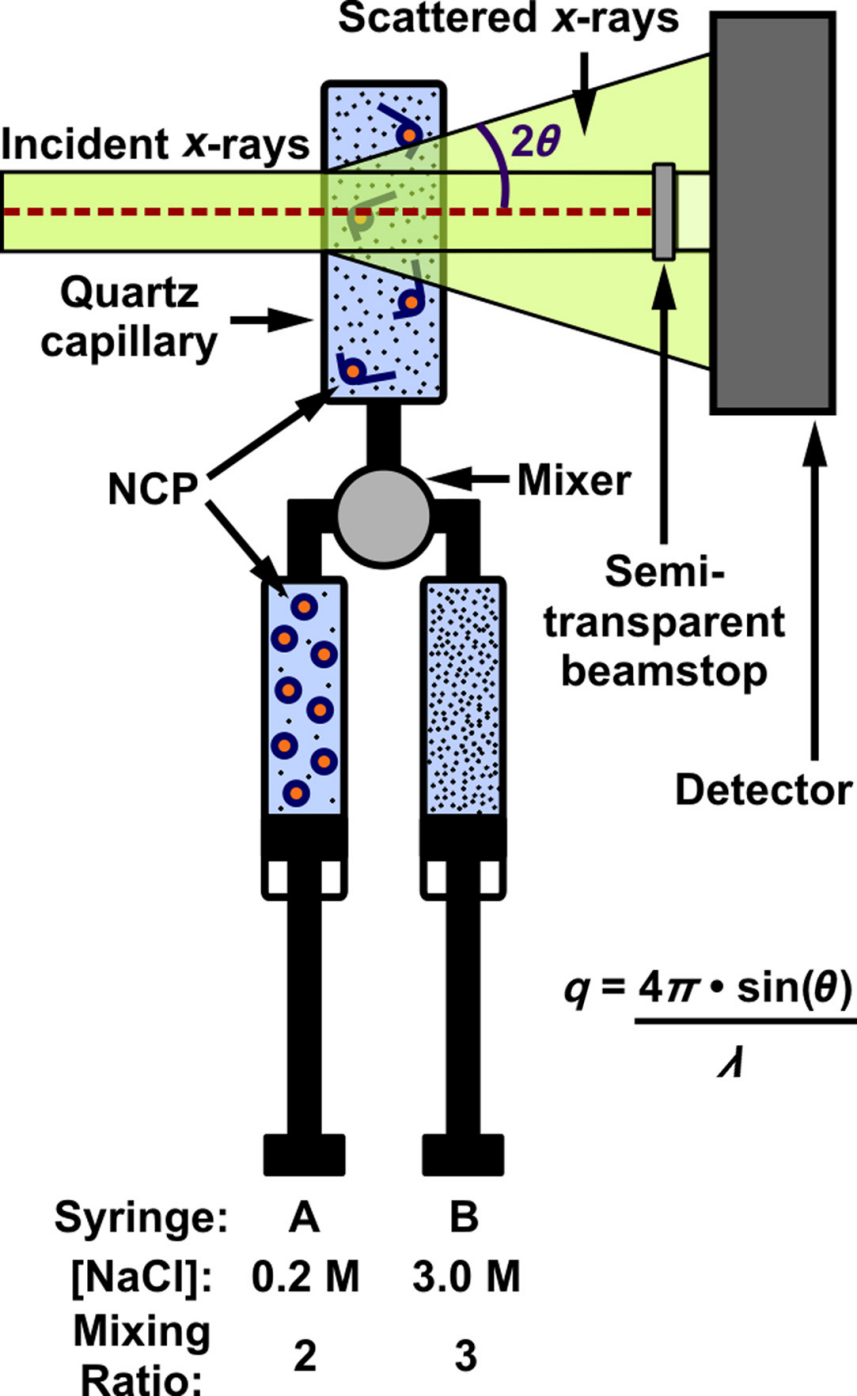
Schematic of stopped-flow mixing experiment to probe salt-induced disassembly of NCPs without sucrose. Compact NCPs in 0.2 M NaCl mix with buffer containing 3.0 M NaCl to achieve a final NaCl concentration of 1.9 M, where full NCP disassembly occurs. The optimal flow rates and volumes used were 6 ml/s and 315 μl for 0% sucrose and 7.5 ml/s and 375 μl for 50% sucrose. In 0% sucrose, both nucleosomal DNA and histones are ‘visible’, hence TR-SAXS data reports changes in NCP global size, structure and composition. In 50% sucrose, only nucleosomal DNA is ‘visible’, TR-SAXS data directly reveals changes in DNA conformation. *λ* is the wavelength of the incident X-rays (in Å).

Small angle X-ray scattering (SAXS) is a label-free technique that reports the global conformation and composition of macromolecules, including NCPs, in solution (21–26). The scattered intensity provides information about the average composition, size and shape of the scattering particles. The extrapolated scattering intensity at zero angle, *I*(0), is proportional to the square of the excess electron density of the particles in solution and is therefore sensitive to changes in the oligomeric state of the complexes. Thus, *I*(0) can be used to monitor the dissociation of proteins from the NCP. A quantitative measure of size is reported as the radius of gyration (*R*_g_). For scatterers with homogenous electron densities, the scattered intensity *I*(*q*) is directly related to macromolecular shape. However, for complexes with components that have varying electron densities (e.g. protein and nucleic acids), the relationship between *I*(*q*) and macromolecular shape becomes ambiguous. The simplest way to circumvent this challenge is to apply contrast variation and match the electron density of solvent with the lower density protein (see Supplementary Text: Contrast Variation). By adding 50% sucrose to the solvent, the protein becomes invisible above the background and only the DNA contributes to the scattering (Figure 1). Contrast variation SAXS has successfully revealed the structure of RNA or DNA complexed with proteins in static studies (27–28). Here we describe the application of contrast variation to monitor changing NCP conformations as [NaCl] is increased in equilibrium titrations. We then expand on this strategy by incorporating a stopped-flow mixer (SFM) to measure time-dependent changes following the rapid addition of salt (Figure 2). Extensive characterization of mixing performance verified a ≈ 5 ms mixing dead time, even for viscous sucrose solutions (see Supplementary Text: Mixer Characterization).

## MATERIALS AND METHODS

### NCP production and reconstitution

The standard procedures used to express and purify *X. laevis* histones are described in Supplementary histone purification. The production of both DNA sequences (Widom 601 and the 5S sequences) is also described in Supplementary DNA production.

Our previous NCP reconstitutions employed size-exclusion HPLC in 2 M KCl to purify histone octamers before the addition of DNA and stepwise dialysis to lower salt concentrations (6). If care is taken to optimize the dimer:tetramer:DNA ratios, NCP preparations of similarly high homogeneity can be obtained without initial purification of the octamer (29). Histone octamers were formed by mixing H2A–H2B and (H3–H4)_2_ in 2 M NaCl, 0.1 mM EDTA and 20 mM Tris–Cl pH 7.5 and incubating on ice overnight before the addition of DNA. Using the button-dialysis method (30), preliminary small-scale reconstitutions were done by step dialysis (2 M to 0.85, 0.65, 0.2 and 0 M NaCl) to determine the optimal conditions for every preparation, varying both the H2A–H2B:(H3–H4)_2_ and octamer:DNA ratios. The quality of the NCP samples was analyzed by 5% native gel PAGE (polyacrylamide gel electrophoresis) (described in detail in (29)) to identify the ratio that resulted in NCPs with no free DNA or other histone–DNA complexes. Subsequent large-scale NCP preparations combined the appropriate amount of histone oligomers and DNA to a final concentration of 5 μM NCP, followed by step dialysis. Samples with the 5S DNA were heat-shifted by incubation at 37°C for 1 h to achieve homogeneously positioned NCPs. NCP samples were concentrated to ∼50 μM by centrifugal ultrafiltration.

### Equilibrium SAXS experiments

SAXS data were collected at the Cornell High Energy Synchrotron Source (CHESS) G1 station with an X-ray energy of 10.5–10.6 keV. Sample-to-detector distance was measured to be ≈1 m using a silver behenate standard. The available *q*-range was from ≈0.007 to 0.25 Å^−1^. Samples and matching buffers were manually prepared and equilibrated for at least 5 min before being loaded into a 2-mm diameter quartz capillary with 10 μm walls (HR6-150, Hampton Research). Samples were oscillated during X-ray exposure to reduce radiation damage. SAXS profiles of matching buffers were measured before and after each sample to monitor beam conditions and ensure capillaries were clean. The scattered X-rays were imaged onto a photon counting array detector (PILATUS 100K, Dectris). Multiple images with 1–30 s exposures were acquired for each sample, and exposure-dependent changes reflecting radiation damage were carefully monitored. Incident beam was measured for normalization using either a PIN diode embedded in the beamstop or a semi-transparent beamstop to directly image the attenuated beam. An NCP concentration series in 0.2 M NaCl showed negligible concentration-dependent inter-particle interactions at ≈5–10 μM. Thus, equilibrium and time-resolved experiments were conducted at these concentrations.

### Time-resolved SAXS experiments

Time resolution was achieved by incorporating a stopped flow mixer (SFM-400, Bio-Logic) as shown in Figure 2. Custom sample cells with 2 mm path length quartz capillaries were used, allowing for direct comparison with equilibrium experiments after adjusting for contrast. NCPs in low salt (0.2 M) were mixed with high salt buffers (3 M) at a ratio of 2:3 to achieve a final NaCl concentration of 1.88 M. Efficient mixing of viscous solutions in the contrast matched condition was ensured by (i) loading both the NCP sample and high salt buffer with 50% sucrose (so the mixing solutions have similar viscosities), (ii) incorporating a high density mixer (model HDS, Bio-Logic) and (iii) optimizing the mixing protocol (see Supplementary Text: Mixer Characterization). Matching buffers were measured before and after each experiment by replacing the sample syringe with low salt buffer.

Several design features were incorporated to maximize the measured SAXS signals and ensure reliable data collection. Background scattering from air and windows was minimized by placing the sample capillary and X-ray flight path under vacuum. A semi-transparent molybdenum beamstop was used to attenuate and image the beam for reliable normalizations. The PILATUS 100K detector was operated in ‘movie mode’ with a time frame of 20 ms (17 ms exposure + 3 ms readout). Because the samples were not oscillated after mixing, the NCPs were susceptible to radiation damage. For samples without sucrose, attenuators were placed in the beam after 10 s to limit radiation damage. Interestingly, samples with 50% sucrose appeared to be less susceptible to radiation damage, thus no attenuators were used.

### Data analysis

All SAXS images were processed using MATLAB (MathWorks). SAXS intensity patterns for each image were azimuthally averaged about the beam center and SAXS profiles from multiple images of the same sample were averaged to improve the signal-to-noise ratio. Uncertainties in *I*(*q*) for each image were estimated as standard deviations divided by the square root of the number of pixels binned for each *q*-value and propagated appropriately. SAXS curves for NCPs were determined by subtracting the scattering curves of matching buffers from the total scattering curves of the samples. Raw data are displayed as Kratky plots, *I*(*q*)·*q*^2^ versus *q*. More globular objects display strong peaks in Kratky plots. Pairwise distance distributions, *P*(*R*), were calculated using the regularized indirect Fourier transform program GNOM (31). High-*q* values were omitted in the fits when they largely affected *P*(*R*) shape in order to avoid artifacts from including data with low signal-to-noise ratio. The largest dimension of the molecule (*D*_max_) was systematically varied until (i) a good fit to the data was achieved, (ii) the *P*(*R*) shape was stabilized and (iii) the *P*(*R*) had a smooth decaying tail. All *I*(0)s and *R*_g_s reported were calculated using GNOM.

For time-resolved studies, the SAXS profiles from the 20 ms exposures were binned to improve the signal-to-noise ratio (at the expense of time resolution). Optimal bin sizes were determined from kinetic Singular Value Decomposition (SVD) analysis (32) (Supplementary Figures S4 and S5). SAXS profiles for corresponding time bins from subsequent experiments (4–6 repeats for each condition) were averaged. *I*(0,*t*), *R*_g_(*t*) and *P*(*R*,*t*) analysis and modeling (see below) for each time point (*t*) were conducted following the same strategies as the equilibrium experiments. In 50% sucrose, SAXS curves extrapolated to nearly identical *I*(*q* = 0,*t*)s, indicating only DNA contributed to the SAXS signal.

### Modeling and *P*(*R*) analysis

Model structures were utilized to gain physical insight into the features observed in *P*(*R*) functions. The DNA component of the crystal structure for the nucleosome core particle (1AOI) was used to model the NCPs in the completely wrapped state. A linear 149 bp DNA using the Widom 601 DNA sequence was generated using Nucleic Acid Builder (33) to model the NCPs in the free unwrapped state. Minor differences in length or sequence identity did not make significant differences. To account for solvation, theoretical scattering profiles were first calculated from the atomic coordinates of the models using the program CRYSOL (34). Theoretical scattering profiles were then processed through GNOM to determine *P*(*R*) and *R*_g_. As shown in Figure 4d, structural features from three length scales were identified. Alternate conformations with the DNA released by varying degrees were generated by appending linear DNA fragments to the DNA component of 1AOI (Supplementary Figure S2). The trends observed in *P*(*R*) as DNA was unwrapped further validate the proposed interpretation of the features (Supplementary Figure S2).

**Figure 3.**
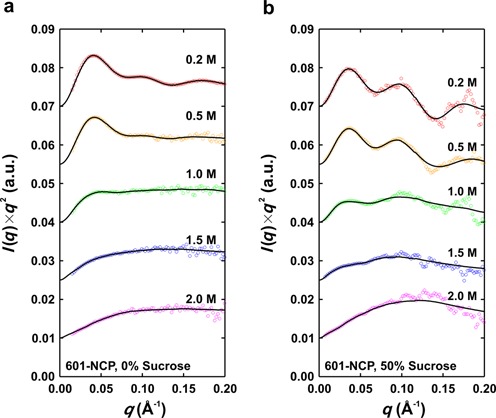
Kratky plots for 601-NCP in varied [NaCl] with (**a**) 0% and (**b**) 50% sucrose. The data (colored circles) and regularized fits to the data (black lines) are scaled and offset to enhance visualization. Because the data in (**b**) are significantly noisier, a moving average with a span of 9 was used to show the quality of the fits. The transition from a compact to an extended structure is observed as the strongly peaked curve changes to a more plateaued curve at high *q* with increasing [NaCl].

**Figure 4.**
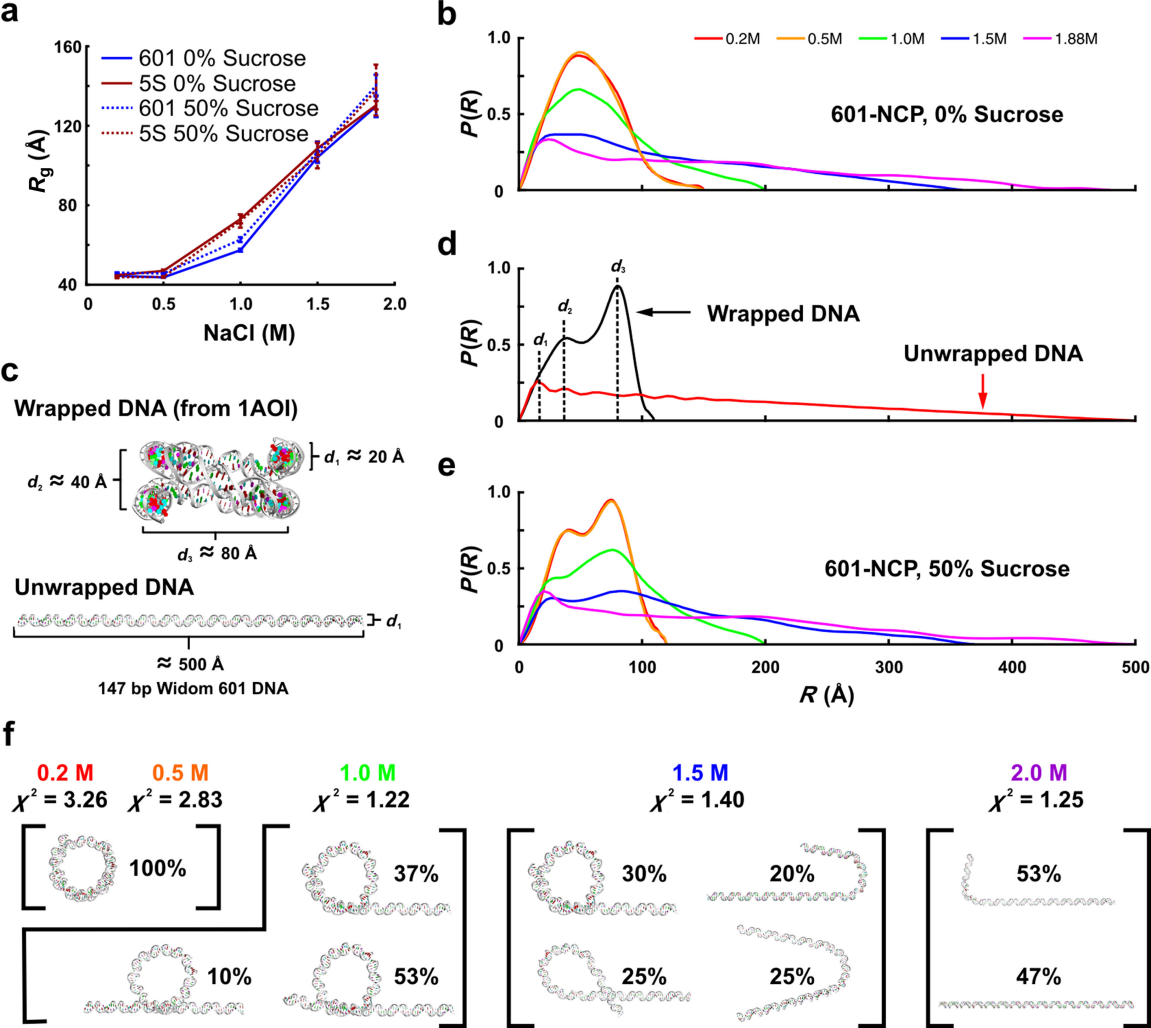
Contrast variation reveals DNA conformation during salt-induced disassembly. (**a**) Radius of gyration (*R*_g_) for 601-NCP and 5S-NCP in equilibrium with different NaCl concentration with and without sucrose. An expansion in size from 45 to 130 Å is detected for both constructs with increasing [NaCl]. At 1 M NaCl and 50% sucrose, the 5S-NCP DNA (72 ± 3 Å) is more expanded than the 601-NCP (63 ± 1 Å). (**b**) *P*(*R*)s for 601-NCP in equilibrium with varied [NaCl] and 0% sucrose. A general extension of the NCP is observed with increasing [NaCl]. (**c**) Models used for calculating theoretical *P*(*R*)s for the wrapped (DNA component from the NCP crystal structure 1AOI) and unwrapped (linear 147 bp Widom 601 DNA) states. (**d**) *P*(*R*) peaks at three length scales are attributed to structural features as follows: *d*_1_—diameter of duplex DNA; *d*_2_—distance between overlapping DNA ends; *d*_3_—diameter of wrapped structure. (**e**) *P*(*R*)s for 601-NCP in equilibrium with varied [NaCl] and 50% sucrose. With the signal from proteins eliminated, *P*(*R*) reveals how DNA conformation changes as the NCP is destabilized by increasing [NaCl]. (**f**) Models representing ensembles of conformations selected by EOM that produce theoretical SAXS profiles that best fit the [NaCl]-dependent SAXS data. Percentages reporting weights of models and *χ*^2^ values assessing overall fit to experimental SAXS data are shown.

## RESULTS

### Equilibrium SAXS reveals salt induced NCP disassembly

As a precursor to time-resolved studies, we first measured the equilibrium response of the NCP to increasing [NaCl], both with and without sucrose (Figure 3 and Supplementary Figure S1). In buffer containing 200 mM NaCl, NCPs are compact with DNA predominantly wrapped around the histone core. At lower ionic strength, repulsive forces are detected between NCPs in the concentration range of interest, leading to inter-particle interference effects that distort the SAXS profiles at the lowest angles (as observed in (35)). Increasing concentrations of NaCl (from 0.2 to 2 M) populate intermediate species similar to those transiently observed at physiological ionic strength (0.05–0.1 M monovalents) (5,12). Depending on the DNA sequence, dissociation of H2A–H2B dimers and the (H3–H4)_2_ tetramer occur above ≈0.6 and ≈1.5 M NaCl, respectively (6,17). At 2.0 M NaCl, the histone proteins are largely dissociated from the DNA. Importantly, FRET-based studies (6) showed that sucrose has no measurable effects on the NaCl-dependent equilibrium stability of NCPs.

**Figure 5.**
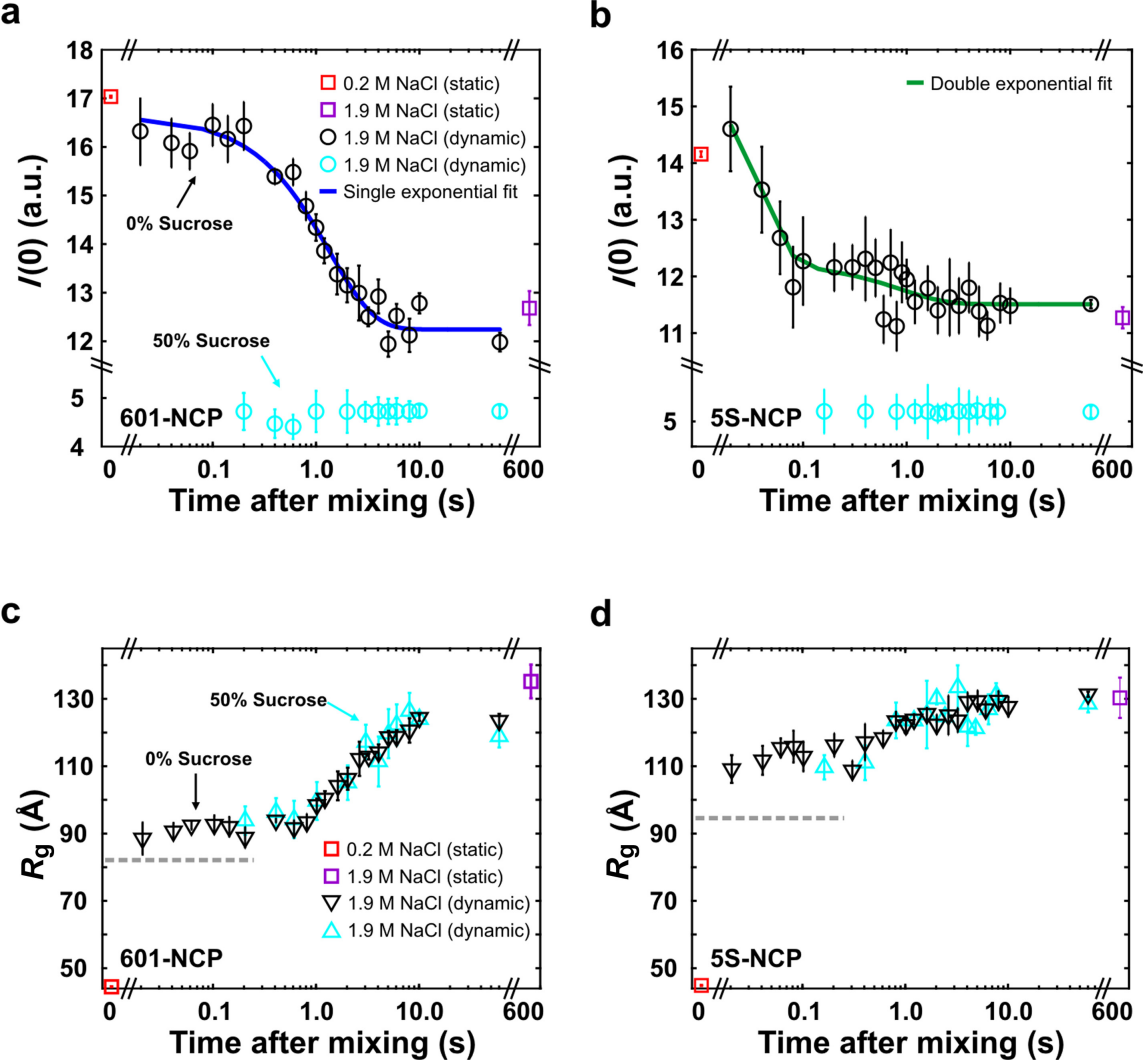
*I*(0,*t*) and *R*_g_(*t*) analysis monitoring protein dissociation and expansion of NCP size as DNA is released. (**a** and **b**) In 0% sucrose, *I*(0) values (black circles) monitor the time-dependent release of histone components for the 601-NCP and 5S-NCP, respectively. The 601-NCP remains intact for the first 200 ms and is described by a single exponential decay, whereas the 5S-NCP appears to dissociate faster, but shows a double exponential decay. In 50% sucrose, *I*(0) values (light/cyan circles) are decreased due to the reduced contrast and remain relatively unchanged since the signal arises from DNA alone. (**c** and **d**) Time-dependent changes in the radius of gyration reveals DNA unwrapping for 601-NCP and 5S-NCP, respectively. Dynamics were monitored on time scales ranging from 20 ms to 60 s after mixing. Equilibrium values for intact (in 0.2 M NaCl, corrected for contrast – see Supplementary Text: *I*(0) Analysis) and fully dissociated (in 2.0 M NaCl for 10 min) NCP states are shown for comparison. The gray dashed lines in (c) and (d) represent the *R*_g_s for 601-NCP (=82.15 Å) and 5S-NCP (=95.37 Å) if the ‘J’-shaped DNAs (Figure 6c and d) are bound to intact histone octamers (models shown in Supplementary Figure S7a).

The [NaCl]-dependent SAXS profiles measured with and without sucrose for the 601- and 5S-NCP constructs show a response to NaCl similar to that reported in previous biophysical assays (5–6,12,17). Raw data, shown as Kratky plots for 601-NCP (Figure 3) and 5S-NCP (Supplementary Figure S1) reveal dramatic conformational transitions from globular to extended structures as the NaCl concentration increases from 0.2 to 2.0 M. The structural details (observed as peaks and troughs in the curves) become significantly more pronounced when the proteins are blanked in sucrose, highlighting the power of contrast variation (Figure 3b and Supplementary Figure S1b). The extension of the NCP with increasing [NaCl] is also reflected by salt-dependent *R*_g_s. (Figure 4a). These raw SAXS data reveal subtle differences between the two constructs. Although the general size for the two constructs is comparable in the compact state at 0.2 M NaCl (43.7 ± 0.5 Å for 601-NCP and 44.9 ± 0.3 Å for 5S-NCP), the broadened peaks and troughs in the Kratky profiles in 50% sucrose suggest a less well-defined structure for the 5S-NCP DNA compared to the 601-NCP DNA (Figure 3 and Supplementary Figure S1). Furthermore, at 1 M NaCl, *R*_g_ values show that the 5S-NCP DNA is more extended than 601-NCP DNA (Figure 4a) suggesting that it is more readily unwrapped by increasing [NaCl]. These differences likely reflect weaker DNA–histone interactions for the 5S sequence relative to 601 (19).

### DNA conformation revealed by *P*(*R*) analysis and ensemble optimization method

The dramatic conformational changes, already revealed in the Kratky plots of Figure 3, are most readily interpreted by examining pairwise distance distribution functions (*P*(*R*)s) computed from SAXS profiles using GNOM (31). Real-space information is displayed in *P*(*R*)s as histograms of all intra-molecular distances *R* (Figure 4b d and e). Peaks in *P*(*R*) represent length scales that are repeated within the particles. The distribution function approaches zero at the largest intra-molecular dimension (*D*_max_). In the absence of sucrose, *P*(*R*) is challenging to interpret because the contributions from protein and DNA components cannot be distinguished. These *P*(*R*)s reveal only general features, such as the overall size and largest dimension of the NCPs (Figure 4b). At low [NaCl], compact NCPs are characterized by *P*(*R*) curves with prominent peaks near 50 Å and a full-width of ∼100 Å, consistent with the largest dimension of wrapped NCPs. Similar *P*(*R*) curves for full NCPs (in 0% sucrose and 0.2 M NaCl) have been reported for several different DNA sequences (22–26). With increasing [NaCl], NCPs are destabilized, and DNA unwrapping is observed as the general extension of the *P*(*R*) curves to ∼500 Å, the length of the free dsDNA in solution.

When sucrose is added and the proteins ‘disappear’, clear and identifiable features emerge in the [NaCl]-dependent *P*(*R*) curves, revealing specific conformational details of the unwrapping DNA (Figure 4e, detailed in Supplementary Figure S2). To interpret the features present in these histograms, we computed the scattering profiles of model structures using the wrapped and unwrapped models of nucleosomal DNA (Figure 4c and d). As the DNA dissociates: (i) a peak appears at *d*_1_ ≈ 20 Å, revealing an extension of DNA duplex, (ii) the peak at *d*_2_ ≈ 40 Å disappears, corresponding to the decrease in overlap between DNA ends and (iii) the peak at *d*_3_ ≈ 80 Å decreases, corresponding to a disruption of the overall wrapped structure. Clearly, contrast variation SAXS provides incisive structural information about DNA conformations accompanying [NaCl]-induced NCP dissociation.

To elucidate the configuration of nucleosome-bound DNA at different NaCl concentrations, we generated a pool of candidate DNA conformations for comparison with the contrast matched data. This pool contains both symmetric and asymmetric structures (representative structures from the pools are shown separately in Supplementary Figure S8). Because multiple states may be present at intermediate NaCl concentrations, the ensemble optimization method (EOM) (36) was used to identify collections of structural models that best recapitulate the [NaCl]-dependent SAXS data (see Supplementary Text: Ensemble Optimization Method). The DNA models shown in Figure 4f were selected by EOM analysis from a pool of 32 models, containing both symmetric and asymmetric structures. In 0.2–0.5 M NaCl, the ensemble is relatively monodisperse, with DNA wrapped around the histone core. As the NaCl concentration increases to 1.0 M, roughly half of the DNA is released, but the DNA ends are still crossed. At 1.5 M NaCl, more of the DNA is released but an increased heterogeneity appears in the chosen models. Finally at 2.0 M NaCl, most of the DNA is released. In the ensembles that represent 1 and 1.5 M NaCl, >75% of the models selected are asymmetric. The relationship between peaks in *P*(*r*) at the three length scales *d*_1_, *d*_2_, *d*_3_ and the structural features of the ensembles are consistent with interpretations based on *P*(*r*) curves computed for the representative model structures (Supplementary Figure S2).

### Time-resolved SAXS reports changes in NCP composition and size during disassembly

To measure the structures of transient states accompanying [NaCl]-dependent NCP dissociation, we coupled SAXS with a stopped flow mixer (Figure 2). Fully wrapped NCPs in 0.2 M NaCl were rapidly mixed with buffer containing 3.0 M NaCl in a 2:3 ratio to achieve a final NaCl concentration of 1.9 M. Time-resolved SAXS measurements were carried out with and without sucrose. Representative curves at different time points after mixing are shown in Supplementary Figure S3a and b. At 0% sucrose, the time-dependent dissociation of histone components from NCPs is revealed by monitoring changes in *I*(0) (21). *I*(0) is related to the molecular mass of NCP, but interpretation of *I*(0) requires knowledge of both sample heterogeneity and contrast. It is important to note that the contrast depends not only on sucrose, but also on [NaCl]. Because a wide range of [NaCl] was used for the equilibrium experiments, we limited our analysis to the endpoints with the assumption of monodispersity (fully associated octamers in 0.2 M NaCl and fully dissociated in 2.0 M NaCl). Because time-dependent changes using a stopped flow mixer are measured against a fixed background, the contrast does not change with time. Relative changes in *I*(0,*t*) reveals details of NCP dissociation.

Figure 5a and b shows *I*(0,*t*) for 601- and 5S-NCPs in 1.9 M NaCl without sucrose. Equilibrium values for the intact and completely disassembled NCPs are shown for comparison. The *I*(0) values have not been concentration corrected, so are arbitrarily scaled for each construct. For both sequences, *I*(0) shows no decrease at the earliest time point measured (20 ms), suggesting that all histone proteins remain bound to nucleosomal DNAs on this rapid time scale. For the 601-NCP, *I*(0) remains constant for the first 200 ms after mixing. Protein dissociation occurs with apparent first-order kinetics and a time constant of 0.74 ± 0.08 s^−1^. Significant variation is seen for 5S-NCPs where the time course of *I*(0) is well-described by a double exponential decay with two distinct rates: 41.6 ± 13.9 and 1.13 ± 0.74 s^−1^. Because the amplitude of the second phase is small, we also analyzed the time-binned SAXS curves using singular value decomposition, which projects each curve onto basis states and provides a sensitive measure of subtle changes (Supplementary Figure S5c, see Supplementary Text: Singular Value Decomposition). Two transitions emerge from this analysis with rates that are similar to those of *I*(0). Although the histone proteins dissociate in a single phase from 601 DNA, the two phase curve of the 5S construct indicates a different pattern of protein dissociation.

To assess the overall extent of the NCP structure, we computed the time-dependent radius of gyration, *R*_g_ (Figure 5c and d), for both constructs and compared the values with those obtained from equilibrium studies for intact (≈45 Å) and fully disassembled (≈130 Å) NCPs. At the earliest measured time point, the *R*_g_s of 90 Å for 601-NCP and 110 Å for 5S-NCP are larger than at 0.2 M NaCl, suggesting a significant expansion within the first 20 ms. Interestingly, for the 601-NCP, plateaus observed in both *I*(0,*t*) and *R*_g_(*t*) suggest a kinetic intermediate that persists for the first ≈200 ms. The unchanging *I*(0) value indicates that all of the histone proteins remain bound, despite expansion to a structure with *D*_max_ ≈ 325 Å. In contrast, the rapidly decreasing *I*(0) values for 5S-NCP reveal that the protein core breaks up and dissociates in less than 0.1 s. By 10 s, both constructs reach a highly expanded size (≈500 Å), with minimal further increase.

### NCP intermediate structures revealed by contrast variation TR-SAXS

In order to integrate structural models from time-resolved data acquired in 0 and 50% sucrose, we first confirmed that sucrose has a minimal effect on the dissociation dynamics. SVD analysis of time-binned SAXS curves with and without sucrose (representative curves are shown in Supplementary Figure S3) showed similar rates, suggesting that sucrose minimally alters NCP dissociation dynamics (Supplementary Figures S4 and S5). Furthermore, time-dependent changes in *R*_g_ are not affected by the viscosity of the sucrose, suggesting that DNA unwrapping is not diffusion driven (Supplementary Figure S7a).

**Figure 6.**
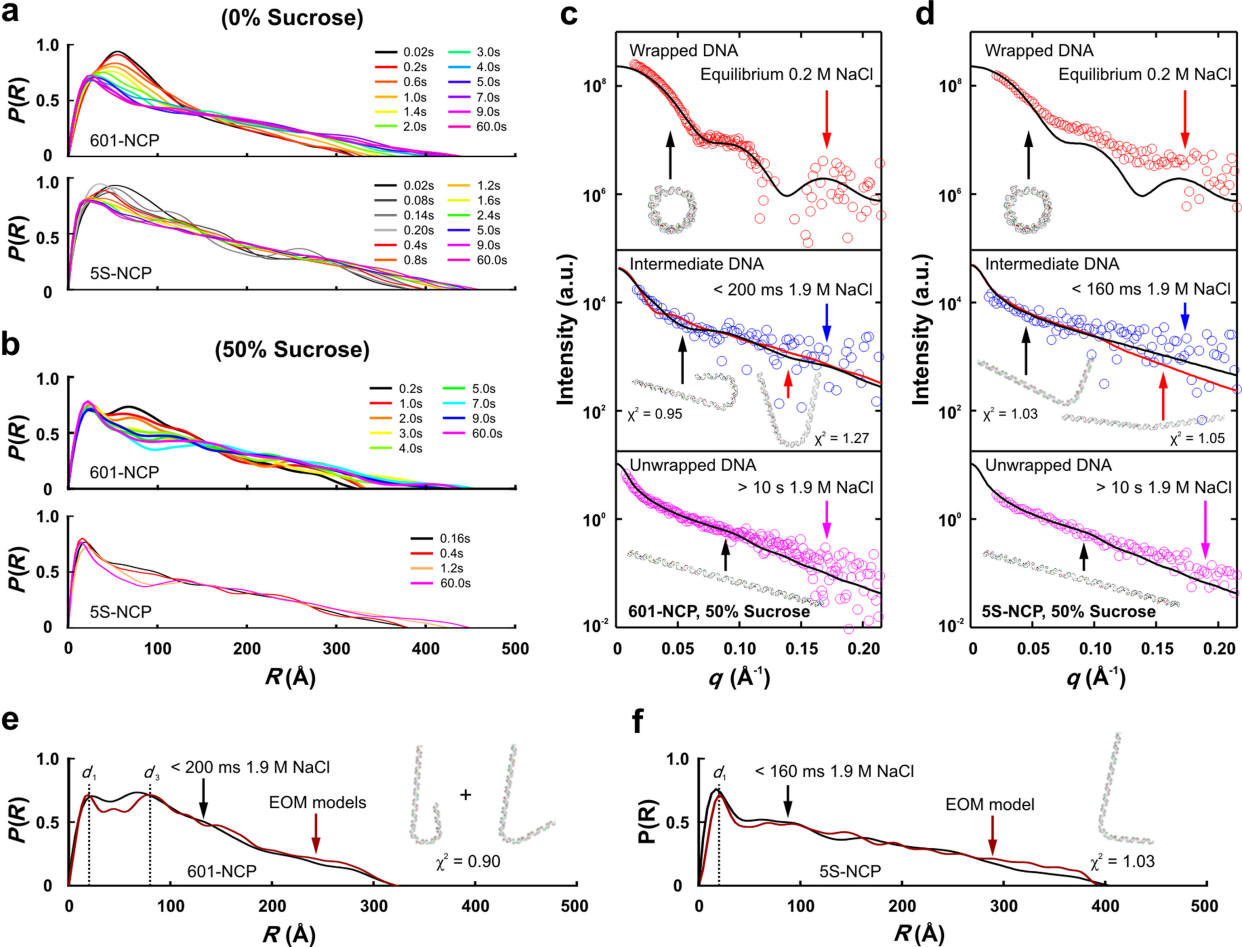
Time-resolved SAXS with contrast variation reveals DNA conformation of kinetic intermediates. (**a**) Pairwise distance distribution functions, *P*(*R*), computed from time-resolved scattering profiles of 601-NCP and 5S-NCP in 0% sucrose. (**b**) Time-resolved *P*(*R*)s for 601-NCP and 5S-NCP in 50% sucrose revealing DNA conformational changes during salt-induced disassembly. (c and d) Comparison of experimental scattering profiles for (**c**) 601-NCP and (**d**) 5S-NCP in 50% sucrose with best fitting theoretical scattering profiles for symmetric (black lines) and asymmetric (red lines) models for the wrapped, intermediate and unwrapped DNAs (offset to aid visualization). Theoretical profiles are calculated from the models shown as insets. The intermediate DNA models were determined using EOM and the goodness of fits was assessed by comparing *χ*^2^ values. (e and f) *P*(*R*)s for the ensembles (red) selected by EOM analysis (models shown with *χ*^2^ fit to SAXS data) compared with the experimental *P*(*R*)s (black) determined from (**e**) the 200 ms kinetic intermediate of the 601-NCP in 50% sucrose and (**f**) 160 ms data of the 5S-NCP in 50% sucrose (for details see Supplementary Text: Minimum Chi-square (*χ*^2^) Fit, Ensemble Optimization Method and Supplementary Figure S8).

Applying the *P*(*R*) analysis to the kinetic scattering profiles, we characterized the dynamic conformational changes of both constructs at 0 and 50% sucrose. In 0% sucrose, both *P*(*R*) distributions at *t* = 20 ms contain a peak similar to that observed in the fully bound NCP, but with *D*_max_ values of 320 Å for 601-NCP and 385 Å for 5S-NCP (Figure 6a and b). At longer times, the major peak position shifts from ≈50 to ≈ 20 Å, characteristic of DNA release observed in static experiments (Figure 4b). These changes occur more rapidly for the 5S-NCP (Figure 6a and b and Supplementary Figure S6a and b) than for the 601-NCP, indicating its decreased stability.

In 50% sucrose, *P*(*R*) analysis provides structural information about the DNA conformation in the 601-NCP intermediate (*t* < 200 ms). Two characteristic peaks appear, at distances *d*_1_ and *d*_3_ (shown in Figure 6e). Comparison with static analysis of Figure 4d and Supplementary Figure S2 suggests that in this state, the DNA has a very large dimension (*D*_max_ ≈ 325 Å), does not overlap itself (missing *d*_2_ peak at ≈40 Å) but nevertheless forms nearly a complete wrap around the histones (a pronounced *d*_3_ peak at ≈80 Å). Based on geometrical arguments, these structural signatures support an asymmetric DNA conformation. A comparison of the experimental data with computations based on symmetric and asymmetric models from a library of candidate DNA structures showed strongest agreement for a ‘J’-shaped asymmetric model. Goodness of fit was assessed by a *χ*^2^ test and ensemble optimization method (Supplementary Figure S8). SAXS profiles and models of DNA release from 601-NCP and 5S-NCP are shown in Figure 6c and d. A similarly detailed kinetic analysis of 5S-NCP dissociation proved challenging due to its more rapid dissociation: the 5S-NCP is almost completely unwrapped on the millisecond timescale. However, *P*(*r*) and EOM analysis of the first 160 ms time-resolved SAXS data of 5S-NCP also support asymmetric release (Figure 6f and Supplementary Figure S8).

Structural models of the 601-NCP kinetic intermediate can be refined by integrating all of the above data. The DNA conformation is asymmetric, all proteins remain associated and the *R*_g_ measured for the 601-NCP intermediate (≈90 Å, Figure 5c) is significantly larger than that computed for models of the NCP with the histone intact as an octamer on the wrapped end (≈82 Å, Supplementary Figure S7a). With the DNA constrained to a ‘J’ shape, this dramatic increase in *R*_g_ is best explained by NCP models where protein–protein interactions are disrupted but protein–DNA interactions are not. All histone proteins remain bound, but the octamer core is no longer integral. Additional support for this model is provided from full scattering profiles (Supplementary Figure S7b).

In contrast, the 5S-NCP exhibits much faster salt-induced dissociation dynamics, with no stable intermediate detected. Despite these differences, it is notable that the 5S DNA appears to unwrap asymmetrically, like the 601 DNA. This assessment is based on *P*(*R*) and EOM analyses of the first 160 ms TR-SAXS curve with 50% sucrose (Figure 6d and f) and suggests that asymmetric release is a common feature among different DNA sequences. The histone core is disrupted before dissociation, based on the *R*_g_ analysis (Figure 5d and Supplementary Figure S7a).

## DISCUSSION

The present study reveals the conformations of nucleosomal DNA in response to increasing concentrations of NaCl. NCP disassembly induced by increasing [NaCl] proceeds through steps that may mimic those observed in the nucleus (5,12). Thus, it is reasonable to hypothesize that the conformational changes of the NCP accessed by varying salt concentrations probe the inherent dynamic properties of the NCP that dictate interactions with cellular machinery during chromatin function.

Representative DNA models that best describe the SAXS data for 601-NCPs in equilibrium with varying [NaCl] are shown in Figure 4f. Interestingly, many of the models selected contain asymmetrically extended DNA. This type of release may reflect the asymmetric affinity of the Widom 601 sequence reported by Chua *et al.* (37). Detailed analysis of the 5S-NCP was less straightforward, possibly due to heterogeneity that arises from multiple translational settings. The application of contrast variation in conjunction with modeling reveals interesting differences between the former sequence, which was engineered for strong binding, and the latter, weaker positioning sequence. Future work with other sequences has the potential to reveal additional dissociation pathways and may elucidate the complex, but very important connections between sequence, spacing and affinity.

**Figure 7.**
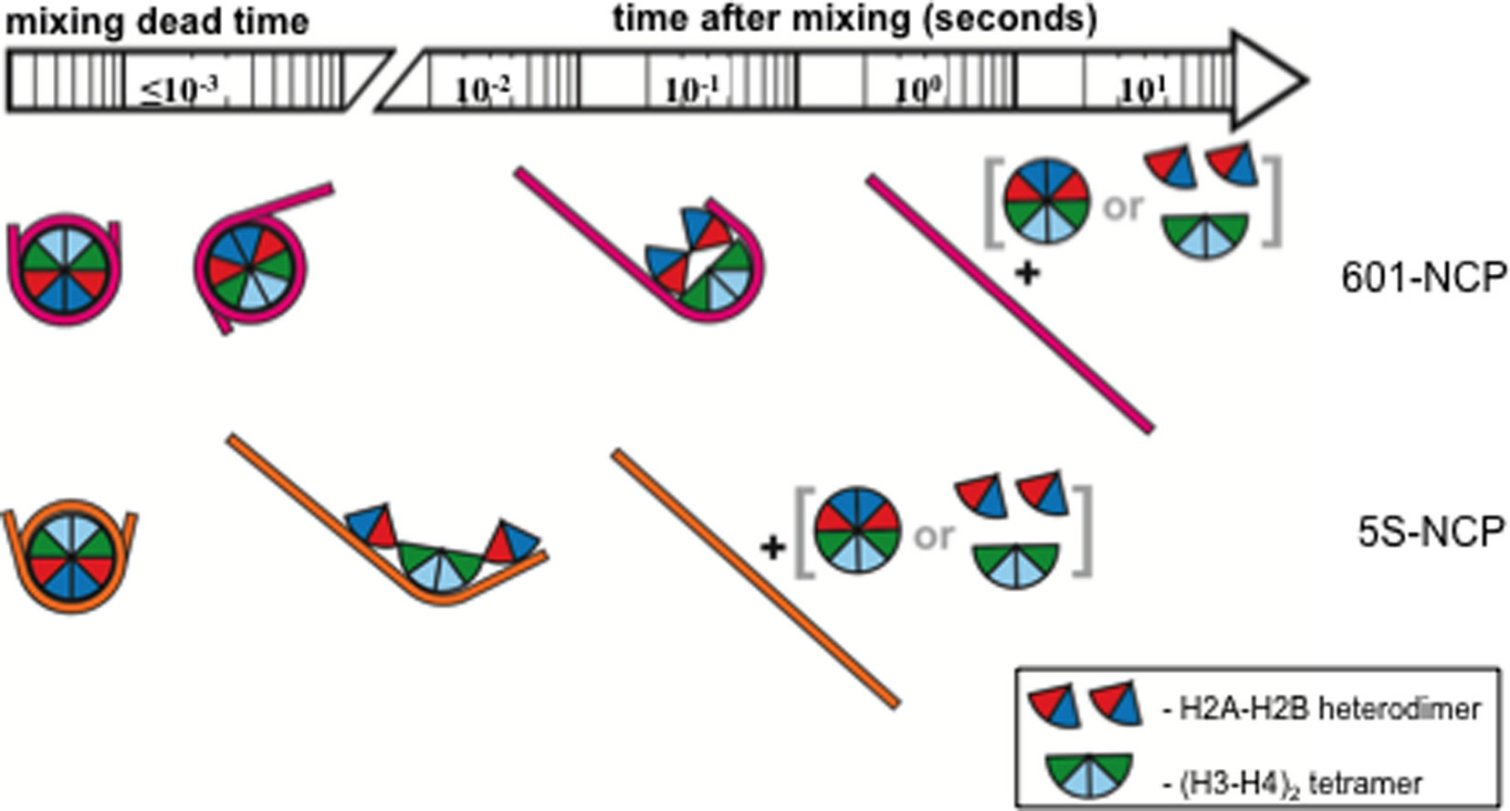
Timeline of salt-induced disassembly of 601-NCP and 5S-NCP. For 601-NCP, DNA opens rapidly from one end and reaches a metastable conformation within the first 20 ms. The histone octamer is disrupted by the asymmetric unwinding, but retains strong electrostatic interactions with 601 DNA and maintains a ‘J’-shaped structure for ∼200 ms. This long-lived intermediate then dissociates at a rate of 0.74 ± 0.08 s^−1^. 5S-NCPs exhibit much faster dissociation dynamics. After 20 ms, the DNA is mostly unwrapped and extended but still bound to the histone components. No stable intermediates are detected and 5S-NCPs disassemble within 1 s (two rates measured: 41.6 ± 13.9 and 1.13 ± 0.74 s^−1^).

Figure 7 presents a schematic timeline for the salt-induced disassembly of the 601-NCP, as well as the less stable 5S-NCP. The novel finding we report is that the early stage of 601-NCP dissociation involves a rapid asymmetric release of the DNA. After about half of the 601 DNA is released, proteins in a disrupted histone core remain trapped in a ‘J’-shaped DNA conformation for at least 200 ms. There is evidence to support the biological relevance of the ‘semi-open’ conformation described here by SAXS and previously by FRET (5). This species may be the preferred substrate for histone chaperones and ATP-dependent chromatin remodeling complexes in transcriptional regulation as well as DNA replication and repair. The population of this intermediate may be regulated by post-translational modifications of the histones or incorporation of histone variants, like H2A.bbd that alter wrapping of DNA (38) or stabilize the NCP to histone exchange, like macroH2A (39). More than 5 s pass before the histones detach from 601 DNA, perhaps a result of strong electrostatic interactions between DNA and the histones. In the 5S NCP, rapid asymmetric DNA release occurs, followed almost immediately by protein dissociation. The absence of a long-lived intermediate precludes a more detailed analysis.

In conclusion, our results establish a powerful platform for studying the global dynamics of nucleosomes and other nucleoprotein complexes that can be triggered by mixing. Technological advancements (i.e. brighter X-ray sources, faster high-viscosity mixers) will soon enable sub-millisecond studies, the timescale of large-scale dynamics for 5S-NCP. The range of potential targets for this technique is broad and includes NCP variants as well as other protein-nucleic acid systems, including RNA–protein machines.

## SUPPLEMENTARY DATA

Supplementary Data are available at NAR Online.

SUPPLEMENTARY DATA
