# Supplementary material for: Revealing transient structures of nucleosomes as DNA unwinds
Source: Nucleic Acids Res. 2014 Jul 1;42(13):8767–76. doi: 10.1093/nar/gku562 (PMC4117781; doi:10.1093/nar/gku562)
Supplement: SUPPLEMENTARY DATA [file supp_42_13_8767__index.html]

Revealing transient structures of nucleosomes as DNA unwinds — SUPPLEMENTARY DATA 

# Revealing transient structures of nucleosomes as DNA unwinds

## SUPPLEMENTARY DATA

**Files in this Data Supplement:**

- Supplementary Data
