# Supplementary material for: Revealing transient structures of nucleosomes as DNA unwinds
Source: Nucleic Acids Res. 2014 Jul 1;42(13):8767–76. doi: 10.1093/nar/gku562 (PMC4117781; doi:10.1093/nar/gku562)
Supplement: SUPPLEMENTARY DATA [file supp_gku562_Chen_Supp_corrected.pdf]

Supplementary Material

Revealing transient structures of nucleosomes as DNA unwinds

Chen, Y. et al.

Supplementary Materials and Methods

Histone Purification

The *Xenopus laevis* histones were expressed recombinantly as inclusion bodies in *E. coli* using previously described T7-expression plasmids[40]. Purification of the individual histones and reconstitution to H2A-H2B and $(H3\text{-}H4)_2$ oligomers was performed as described previously[41-42], except for the final chromatography step over a heparin column, described previously for H2A-H2B purification. First, this step was added to the purification of $(H3\text{-}H4)_2$ as well. Secondly, the chromatography buffers were altered to Tris and NaCl (rather than potassium phosphate and KCl) so that the histones were eluted in similar conditions to those employed in NCP reconstitution. Protein concentrations were determined by absorbance at 280 nm with extinction coefficients of 10,240 and 17,920 $M^{-1}cm^{-1}$ for the H2A-H2B dimer and $(H3\text{-}H4)_2$ tetramer, respectively. The [NaCl] was determined by refractive index.

DNA Production

A plasmid with 24 tandem repeats of the central 149 bp of the Widom 601 DNA was described previously[6], constructed using the iterative approach of ref. 29. For consistency in comparing SAXS data with different DNA sequences, a similar plasmid was constructed for the nucleosome positioning element of the *Lytechinus variegatus* 5S rRNA gene and promoter. The central 144 bp positioning element was PCR-amplified from the pSL208-12 plasmid (a gift from Dr. Michael J. Smerdon's lab, Washington State University) with primers that introduced EcoRV restriction sites at the ends of the PCR product. This fragment was used to engineer a plasmid with 24 tandem repeats. Subsequent plasmid purification, EcoRV digestion to liberate 149 bp fragments and their purification was carried out by protocols detailed elsewhere[6,29]. 601-DNA sequence: [ATCGG AGAAT CCCGG TGCCG AGGCC GCTCA ATTGG TCGTA GACAG CTCTA GCACC GCTTA AACGC ACGTA CGCGC TGTCC CCCGC GTTTT AACCG CCAAG GGGAT TACTC CCTAG TCTCC AGGCA CGTGT CAGAT ATATA CATCC CGAT]. 5S-DNA sequence: [ATCTC CAGGG ATTTA TAAGC CGATG ACGTC ATAAC ATCCC TGACC CTTTA AATAG CTTAA CTTTC ATCAA GCAAG AGCCT ACGAC CATAC CATGC TGAAT ATACC GGTTC TCGTC CGATC ACCGA AGTCA AGCAG CATAG GGCTC GGAT].

Contrast Variation

For SAXS, x-ray photons scatter off particles in solution with excess electron density $\Delta\rho_p = \rho_p - \rho_s$, called contrast, where ρ_p and ρ_s are the average electron densities of the particle and solvent, respectively. This contrast generates a scattering pattern that is distinct from the uniform background scattering of the buffer. For a biomacromolecule, both the overall shape and internal structure contributes to the scattering. However, both the protein and nucleic acid in a complex can be treated as homogeneous components since the difference in average electron densities between the two components is much greater than the electron density fluctuations within each component. Thus, the scattering intensity as a function of contrast for a protein-nucleic acid (PNA) complex can be simplified as[21]

$$I(q) = (\Delta\rho_1)^2 I_1(q) + 2\Delta\rho_1\Delta\rho_2 I_{12}(q) + (\Delta\rho_2)^2 I_2(q)\ , \qquad \text{(\textbf{Supplementary Equation 1})}$$

where $\Delta\rho_1$,$I_1(q)$ and $\Delta\rho_2$, $I_2(q)$ represent the contrasts and scattering intensities of the protein and nucleic acid, respectively. $I_{12}(q)$ is the cross-term intensity.

By increasing the solvent electron density ρ_s to match that of the protein ρ_1, the first and second terms in Supplementary Eq. 1 vanish and the resulting scattering profile is dominated by the nucleic acid component (the third term). Consequently, scattering data containing information about the conformation of nucleic acids in protein complexes can be isolated. The addition of 50% (w/v) sucrose was found to be sufficient to blank histone proteins. This concentration of sucrose had no effect on

the NaCl-dependent equilibrium stability of the NCP, as monitored by a previously described FRET system[6]. The following table shows the average electron densities for biomolecules and the solutions used[21].

**Average electron densities**	
Component	ρ (e^-/nm^3)
Water	334
50% Sucrose (w/v)	400
Proteins	420
Nucleic Acids	550

note: additional molecules (e.g. salts, buffers) in solution also contribute to the actual value

The contrast dependence of any parameters measured in SAXS must be taken into account. For SAXS experiments with sufficiently high contrast, e.g. when using buffers without sucrose or high salt concentrations, these effects are typically negligible.

I(0) Analysis

In SAXS, the average mass of the scattering particles is related to the extrapolated scattering intensity at zero scattering angle I(q = 0). Interpretation of I(0) requires knowledge of both sample heterogeneity and contrast. It is important to note that the contrast depends not only on sucrose, but also on [NaCl]. Since a wide range of [NaCl] was used for the equilibrium experiments, we limited our analysis to the endpoints with the assumption of monodispersity (fully associated octamers in 0.2 M NaCl and fully dissociated in 2.0 M NaCl). The static and kinetic data were measured at the same sample concentrations, allowing for relative comparison of their I(0)s (**Fig. 5a,b**). To take into account the contrast difference between 0.2 M and 1.9 M [NaCl], we applied a scaling factor for the 0.2 M NaCl I(0). This factor was determined through CRYSOL[32] by calculating theoretical I(0)s at two different solvent electron densities for the same wrapped structure (2 M NaCl adds ≈ 30 e^-/nm^3 to pure water). Since time dependent changes using a stopped flow mixer are measured against a fixed background, the contrast does not change with time. Consequently, changes in I(0) report changes in the molecular mass and association state of the proteins as the nucleosomes disassemble. Because multiple association states may be present, we restricted our analysis to the endpoints— which agreed well with the equilibrium results (**Fig. 5a,b**).

Radius of gyration analysis

The radius of gyration (Rg) reported in SAXS is the root mean square distance between the scattering electrons within the particle. The apparent Rg and its contrast dependence is represented as[21]

$$Rg^2 = {R_c}^2 + \frac{\alpha}{\Delta\rho} - \frac{\beta}{(\Delta\rho)^2} \quad , \qquad \text{(\textbf{Supplementary Equation 2})}$$

where R_c is the radius of gyration observed for particles at infinite contrast, Δρ is the contrast, and α and β are parameters that reflect the electron density fluctuations for particles in solution. α characterizes the internal structure of a particle. For a core-shell particle, α is positive when the shell has higher electron density than the core, and negative in the opposite case. β is always positive and increases as the apparent center of mass for one component is displaced relative to the other as a function of contrast.

Rgs reported in this work were determined using GNOM since the analysis uses the entire scattering curve and is less susceptible to low-q noise (in contrast to Guinier analysis). The contrast dependent contributions to Rg (second and third terms in **Supplementary Eq. 2**) were found to be negligible for the range of contrasts involved in this study. Theoretical Rgs determined from NCP models with the solvent electron densities varied from 334-430 e^-/nm^3 showed less than ± 1 Å deviations to the apparent Rgs.

Singular Value Decomposition

Singular value decomposition (SVD) is a powerful strategy for determining the minimum number of components necessary to reconstruct a data set collected over a series of experimental conditions (e.g. over time or a range of NaCl conditions). A set of data plotted with *m* discrete momentum transfer values and *n* scattering curves is arranged as an *m x n* matrix *A*, where each column represents the scattering profile collected within an experimental series. An SVD algorithm (MATLAB) identifies orthogonal basis curves by representing the matrix as a product of three matrices,

$$A = UWV^T \ . \qquad \text{(\textbf{Supplementary Equation 3})}$$

U is an *m x n* matrix of orthogonal columns that form a complete set of basis curves through which the entire series of scattering profiles can be represented by linear superposition. *W* is an *n x n* diagonal matrix containing the *singular values* conventionally ordered by decreasing value. Each singular value w_j represents the overall weight of the basis component U_j. V^T is the transpose of an *n x n* matrix *V*, where columns V_j represents the dependence of columns U_j on the course of the series. Therefore, each column in WV^T contains the linear superposition coefficients for the basis curves at each point in the series. The basis components that make significant signal contributions can be determined by direct inspection and by comparing the corresponding singular values. Although the number of distinctly scattering species corresponds with the number of basis components, the basis components themselves do not necessarily represent morphological SAXS profiles. Independent SAXS curves are linear combinations of the basis components and can be approximated using the independent basis curves U_j(q) as

$$I(q) = \sum_{j=1}^{r} U_j(q) w_j V_j \ , \qquad \text{(\textbf{Supplementary Equation 4})}$$

where *r* is the effective rank, or number of independent basis curves necessary to reconstruct the dataset.

Mixer Characterization

Mixing was characterized through fluorescence assays using N-acetyltryptophanamide (NATA) fluorescence quenching by N-bromosuccinimide (NBS) and Alexa350 quenching by KI . The mixing dead time was measured to be ≈ 6.6 ms using NATA-NBS at a flow rate of 6 mL/s . Incorporation of the HDS mixer reduced convection and back flow, allowing access to timescales up to 60 s. The optimal flow rates and volumes used were 6 mL/s and 315 µL for 0% sucrose and 7.5 mL/s and 375 µL for 50% sucrose. For SAXS experiments, complete salt mixing was confirmed by monitoring the buffer scattering. Mixing between subsequent shots was found to be consistent.

Minimum chi-square ($\overline{\chi^2}$) fit

Agreement between the experimentally observed 601-NCP kinetic intermediate and potential conformational models (**Supplementary Fig. 8**) was first assessed by evaluating the following chi-square:

$$\overline{\chi^2} = \frac{1}{M} \sum_{i=1}^{M} \left(\frac{I_{\text{exp}}(q_i) - I_{\text{model}}(q_i)}{\sigma_{\text{exp}}(q_i)} \right)^2, \qquad \text{(\textbf{Supplementary Equation 5})}$$

where $I_{\text{exp}}(q_i)$ is the experimental scattering intensity at q_i, $I_{\text{model}}(q_i)$ is the scattering intensity calculated from PDB models using CRYSOL, $\sigma_{\text{exp}}(q_i)$ is the experimental error and M is the number of data points in q space. The best fit is revealed by a minimal $\overline{\chi^2}$ value, where a $\overline{\chi^2}$ value less than 1 indicates good agreement. The q-range used was limited to 0.015-0.12 $Å^{-1}$ to reduce the effects of high-q noise.

Ensemble Optimization Method (EOM)

To further investigate possible polydispersity and structural fluctuation, we applied the program GAJOE (Genetic Algorithm Judging Optimization of Ensembles) to determine an ensemble of DNA conformational models, whose combined theoretical scattering intensity best describes the experimental SAXS data (e.g. that of the 601-NCP kinetic intermediate). GAJOE uses a genetic algorithm described in ref. 36. The q-range for GAJOE fitting was also limited to 0.015-0.12 $Å^{-1}$ to reduce the effects of high-q noise. When comparing to the 601-NCP intermediate (observed in the

first 200 ms), one optimized ensemble was first generated from a pool of DNA PDB models (Group 1, 2 and 3 in **Supplementary Fig. 8**), and was found to be populated with mostly asymmetric models. Hence, 20 more asymmetric models (group 4) structurally similar to the most picked model in the first round (the 'Long 80bp released, Short 20bp released' model) were added to the pool, and a new process of ensemble optimization was conducted. The final optimized ensemble contains only similar asymmetric models (**Supplementary Fig. 8** and **Fig. 6e**), indicative of a nearly homogenous intermediary state for the 601-NCP. The $\overline{\chi^2}$ value of 0.90 is also lower than that minimized from single model $\overline{\chi^2}$ fit (0.95 for the 'Long 80bp released, Short 20bp released' model), indicating a better agreement to the experimental measurement when ensemble optimization method is used.

Supplementary Figures

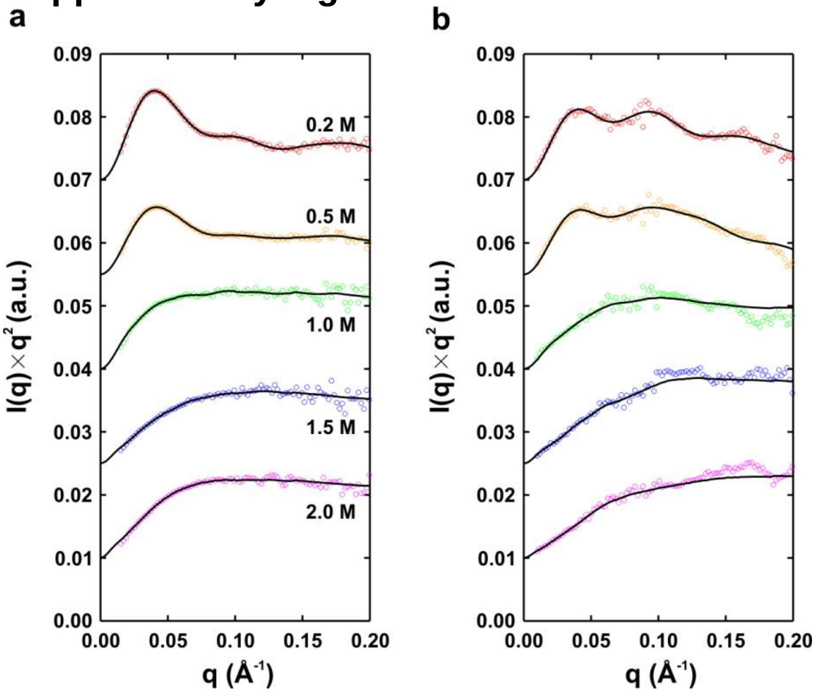

Supplementary Figure 1 Kratky plots for 5S-NCP at different [NaCl] with (**a**) 0% and (**b**) 50% sucrose. The data (colored circles) and regularized fits to the data using GNOM (black lines) were scaled and offset to enhance visualization. A general transition from a globular to an extended structure is observed as the NaCl concentration was increased from 0.2 M to 2.0 M. Many more structural details (visualized as peaks and troughs) are distinguishable with the proteins blanked in (**b**) 50% sucrose compared to the (**a**) 0% sucrose data. Interestingly, the peaks for the 5S-NCP in low NaCl were generally more broadened compared to the 601-NCP (**Fig. 3a**,**b**), reflecting greater conformational variation. Furthermore, the 5S-NCP appears mostly unwrapped at 1 M NaCl, suggesting that 5S-NCP is less stable than 601-NCP. Plots were scaled and offset to enhance visualization.

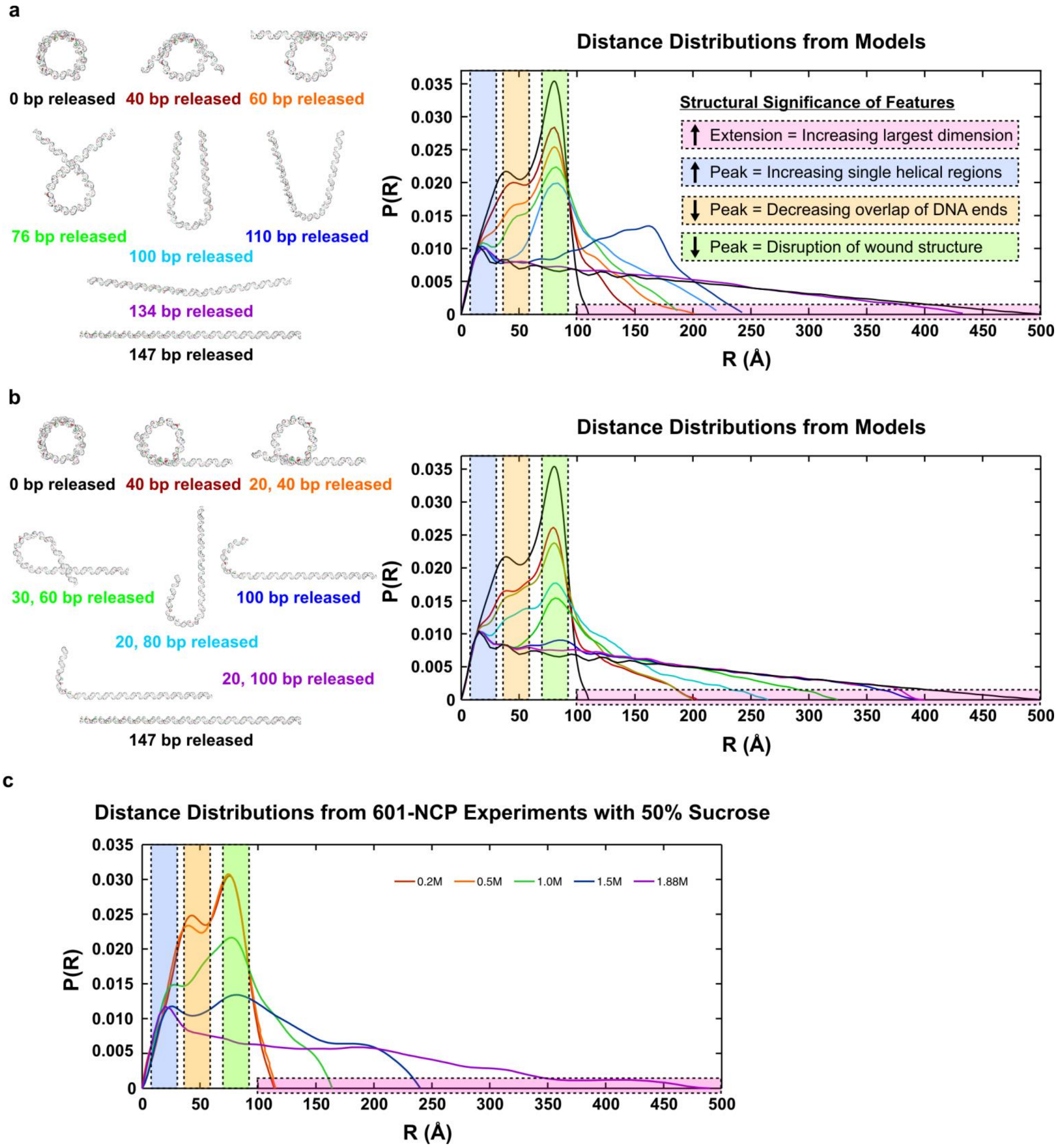

Supplementary Figure 2 Comparison of P(R) curves calculated from models and equilibrium 601-NCP experiments. (**a**) Models with symmetrically released DNA from crystal structure 1AOI and theoretical P(R) curves for models shown. Four regions that provide physical insight into the conformational changes as the DNA is unwrapped are highlighted with colored boxes. The region in pink highlights the extension of the P(R) curves to the right that corresponds with the increasing largest dimension of the models. The region in blue reveals a formation of a peak at approximately 20 Å that corresponds with increasing single-helical extensions. The region in orange shows a decreasing peak at approximately 40 Å that reflects the decreasing overlap between the DNA ends (e.g. compare green to blue curves/models). The region in green shows a decreasing peak at approximately 80 Å that reflects the disruption of the overall wrapped structure. (**b**) Models with asymmetrically released DNA from crystal structure 1AOI and theoretical P(R) curves for models shown. (**c**) Experimental P(R) curves for 601-NCP in 50% sucrose and varying NaCl concentrations. Increasing the NaCl concentration induces conformational changes that reflect the same changes observed in the models.

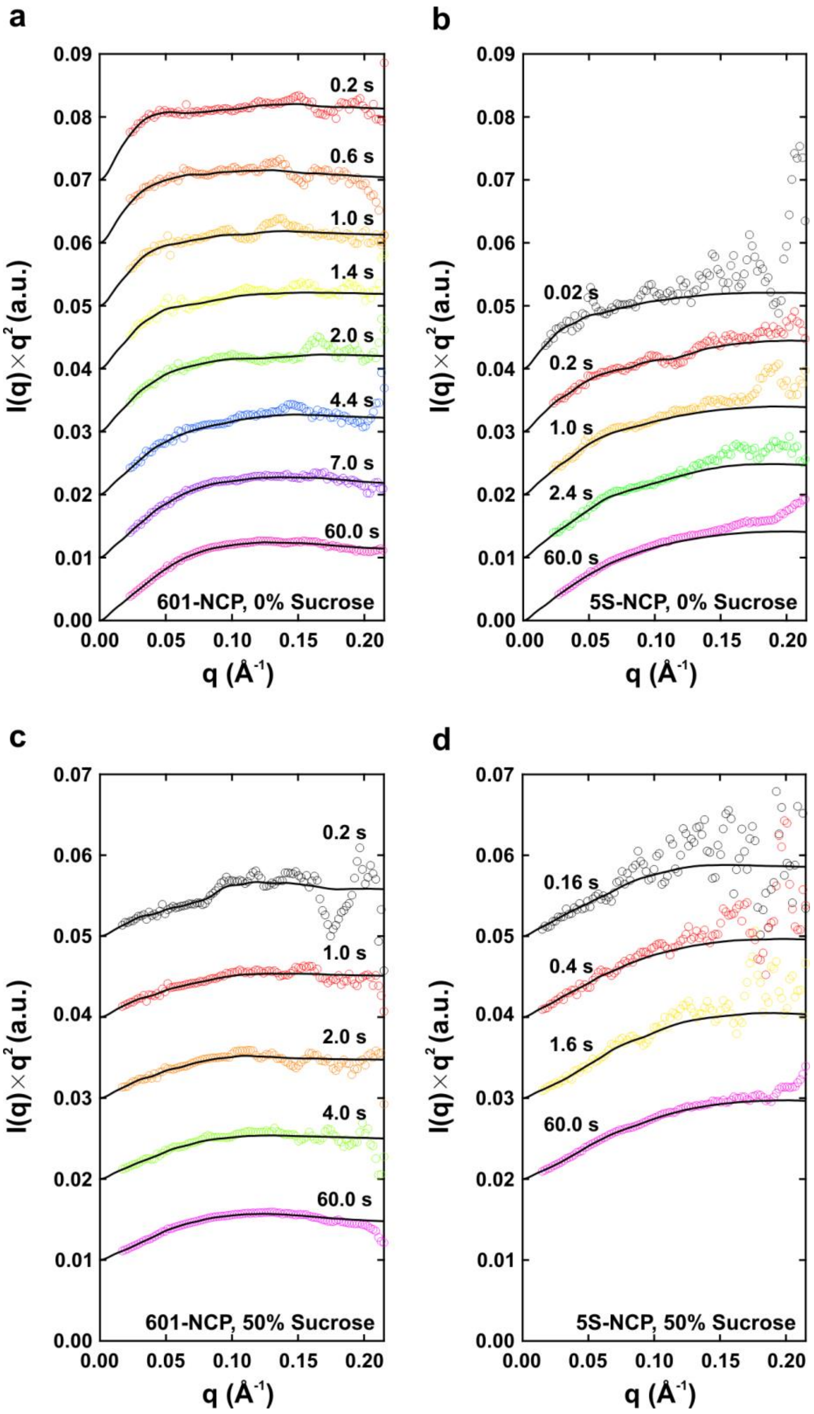

Supplementary Figure 3 Time-resolved SAXS data for 601-NCP and 5S-NCP with and without sucrose. (**a,b**) Kratky plots ($I(q)xq^2$ vs. q) for NCPs dissociating in 1.9 M NaCl with 0% sucrose (protein and DNA visible) at the times indicated after mixing. For both (**a**) 601-NCP and (**b**) 5S-NCP, experimental data are shown as colored circles and regularized fits computed by GNOM are shown as solid black curves. Data has been offset to enhance visualization. (**c,d**) Kratky plots for NCPs dissociating in 1.9 M NaCl with 50% sucrose (only DNA visible) at the times indicated after mixing. For both (**c**) 601-NCP and (**d**) 5S-NCP, experimental data and fits are shown as colored circles and solid black lines, respectively, and the data has been offset to enhance visualization.

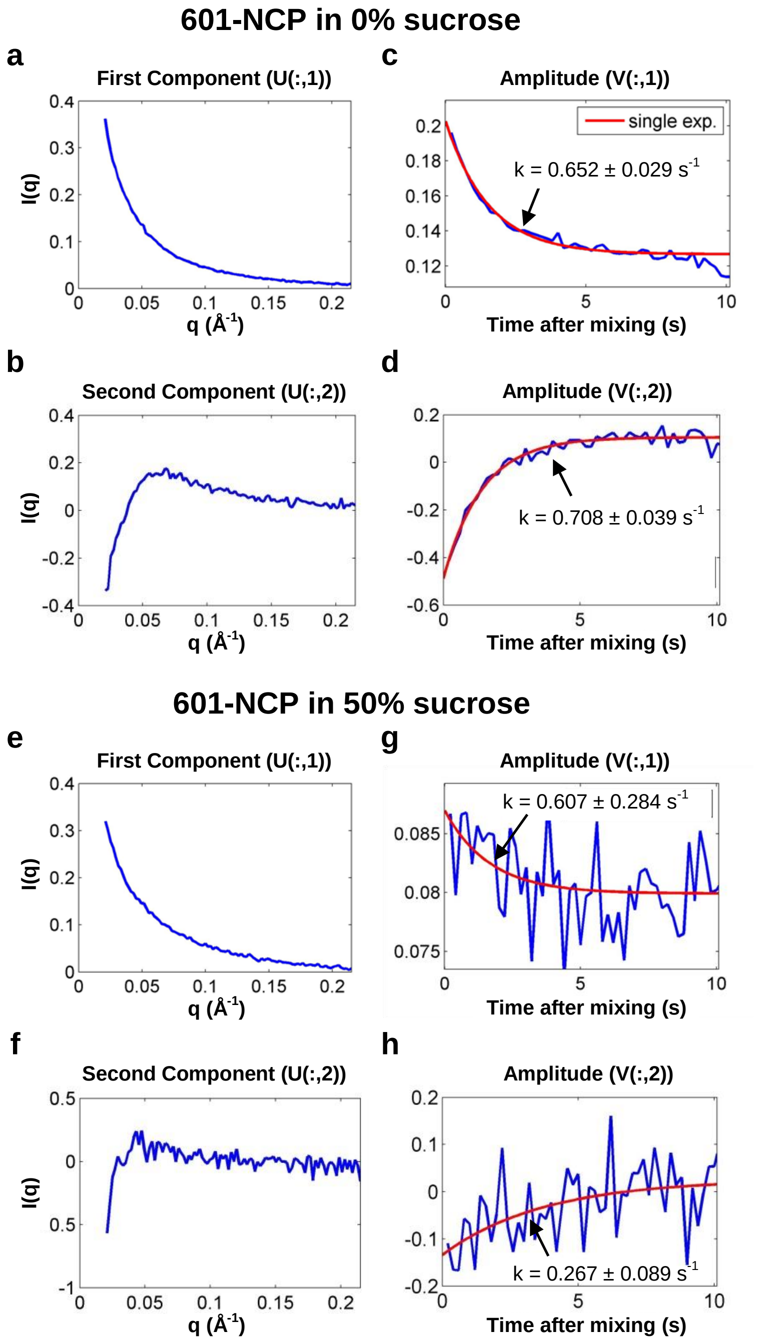

Supplementary Figure 4 SVD analysis of TR-SAXS curves for 601-NCP in (**a-d**) 0% sucrose and (**e-h**) 50% sucrose. (**a,e**) The first basis components determined by SVD analysis of time binned SAXS curves. SVD analysis for time-resolved data was limited to regular linear profiles since TR-SAXS data has significantly more noise than static data. The first basis components dominate the other components by more than one order of magnitude. (**b,f**) The second basis components determined by SVD analysis of time binned SAXS curves. (**c,d,g,h**) Amplitudes of the components in (**a,b,e,f**) as a function of time after mixing. Because the first components dominate the SAXS profile changes, the amplitude changes (**c,g**) of the first components (**a,e**) correspond with the major transition (representing NCP disassembly). The time courses are well described by single exponential decay functions. Their nearly identical rates (k) between the sucrose and non-sucrose conditions indicate that sucrose has minimal effects on NCP dissociation dynamics. Analysis beyond the extraction of time constants from the major changes in the linear profiles was limited by data quality.

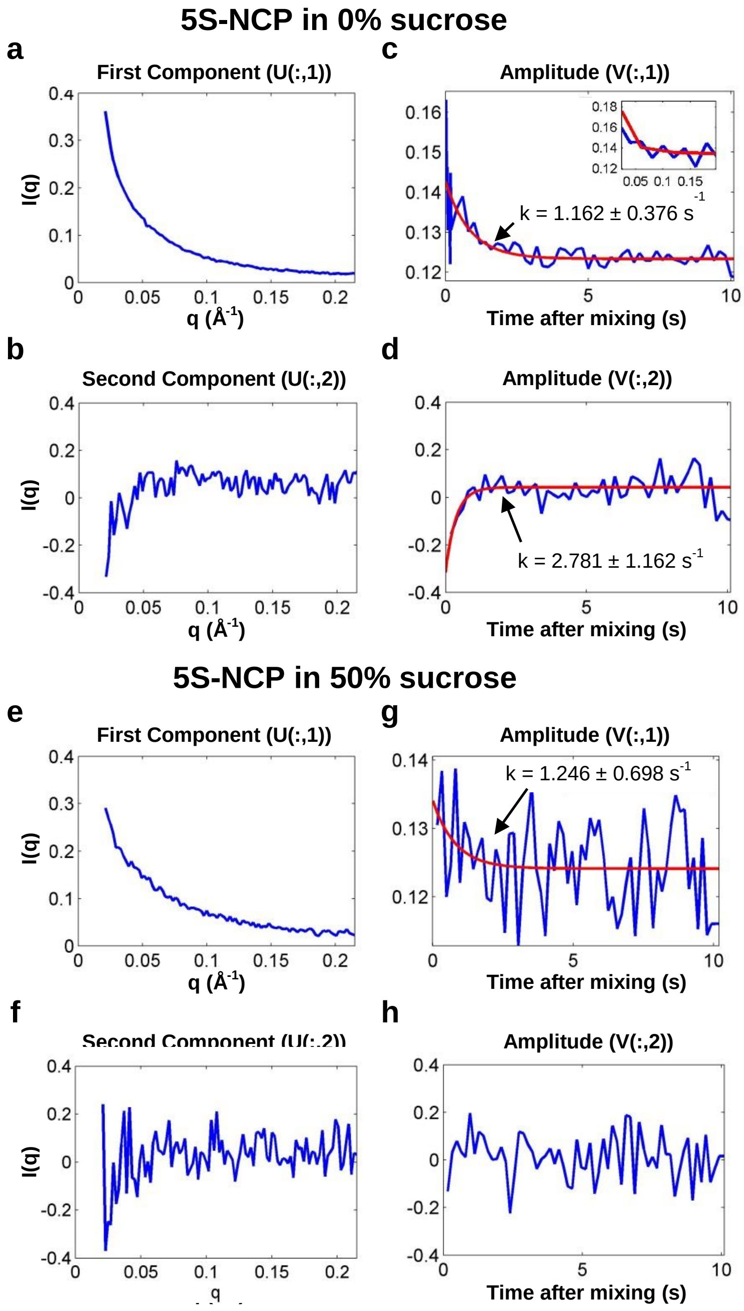

Supplementary Figure 5 SVD analysis of TR-SAXS curves for 5S-NCP in (**a-d**) 0% sucrose and (**e-h**) 50% sucrose. (**a,e**) The first basis components determined by SVD analysis of time binned SAXS curves. SVD analysis for time-resolved data was limited to regular linear profiles since TR-SAXS data has significantly more noise than static data. The first basis components dominate the other components by more than one order of magnitude. (**b,f**) The second basis components determined by SVD analysis of time binned SAXS curves. (**c,d,g,h**) Amplitudes of the components in (**a,b,e,f**) as a function of time after mixing. Because the first components dominate the SAXS profile changes, the amplitude changes (**c,g**) of the first components (**a,e**) correspond with the major transition (representing NCP disassembly). The time courses are well described by single exponential decay functions. Their nearly identical rates (k) between the sucrose and non-sucrose conditions indicate that sucrose has minimal effects on NCP dissociation dynamics. Inset for (**c**): a fast phase of the amplitude change of the first component for 5S-NCP without sucrose. Analysis beyond the extraction of time constants from the major changes in the linear profiles was limited by data quality.

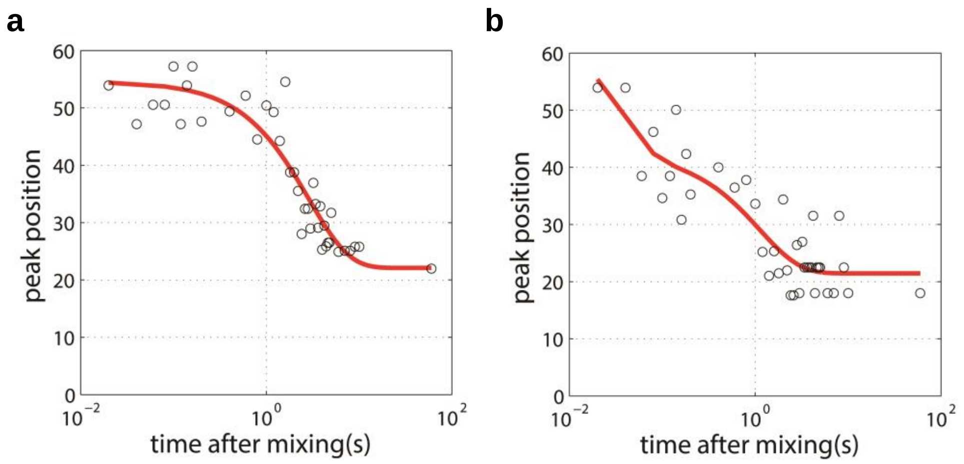

Supplementary Figure 6 Major length scale analysis for (**a**) 601-NCP and (**b**) 5S-NCP in 0% sucrose**.** The position of the major peak in P(R) is plotted as a function of time. (**a**) 601-NCP, a single exponential fit yields a rate of 0.348 ± 0.082 s^{-1}. (**b**) 5S-NCP, a double exponential fit yields two rates: 36.4 ± 18.6 s^{-1} and 0.901 ± 0.354 s^{-1}. The fast phase observed for 5S-NCP without sucrose observed in SVD analysis (**Supplementary Figure 5c**) agrees with that observed in the major length scale analysis (**b**) as well as I(0,t) analysis (**Fig. 5b**).

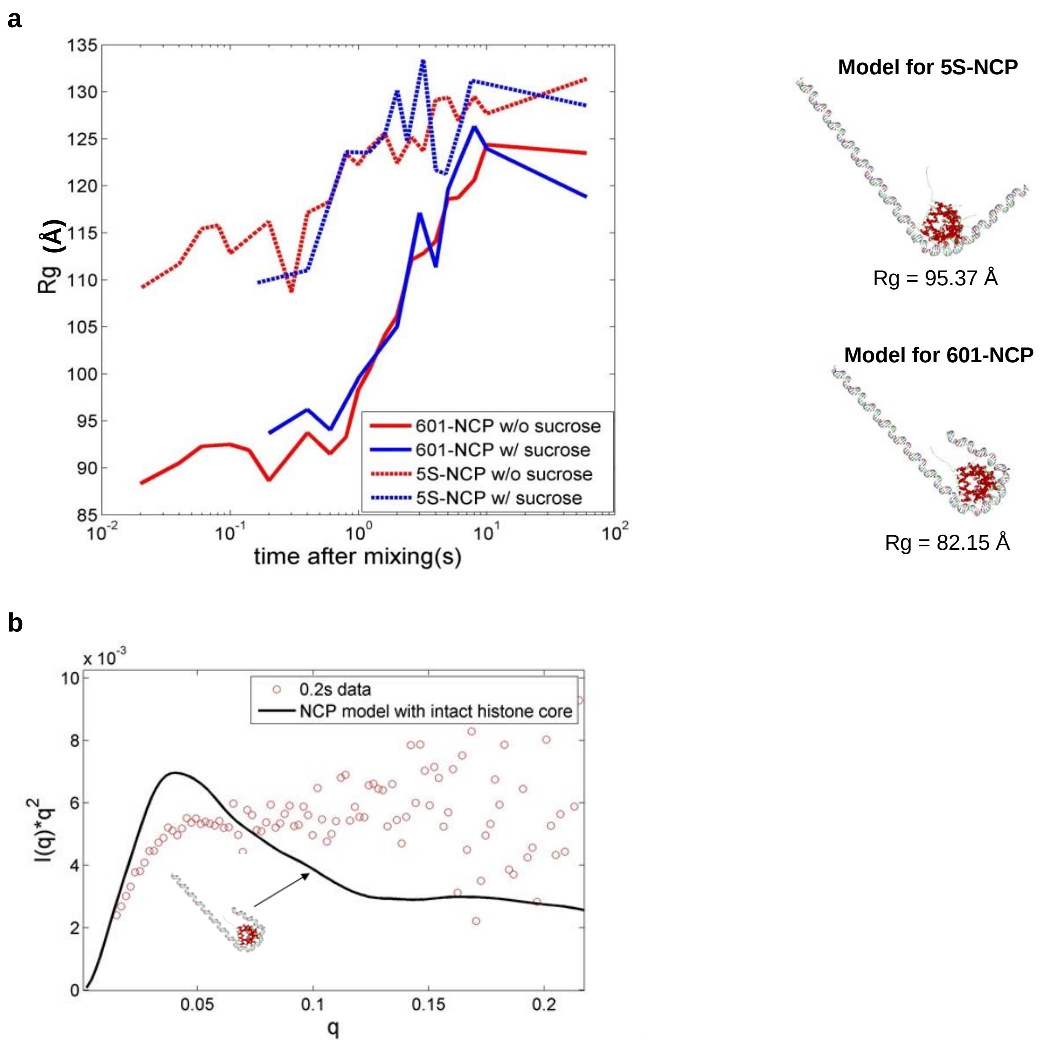

Supplementary Figure 7 Rg and Kratky evidence for a disrupted histone core. (**a**) Rg(t) of 601-NCP and 5S-NCP in 50% and 0% sucrose. For 601-NCP, the majority of the histone proteins dissociate after ≈ 1 s (**Fig. 5a**). As a result, the DNA becomes the dominant scattering particle and Rg(t)s are quite similar between 0% and 50% sucrose. This was also observed for the 5S-NCP, except the majority of the histone proteins dissociate within the first ≈ 100 ms (**Fig. 5b**). The 5S-NCP is generally more extended compared to the 601-NCP through the timescales observed. The Rgs for the 601-NCP 200 ms kinetic intermediate (Rg ≈ 90 Å) and 5S-NCP 160 ms ensemble average (Rg ≈ 112 Å) without sucrose are significantly larger than that computed for models of the NCP as shown in the two models on the right. The DNA structures in the two models were selected by single model chi square fits to the SAXS data and insight provided by P(R) analysis (**Supplementary Fig. 8 and Fig. 6**). The models with the histone intact as an octamer on the wrapped end of the J-shaped DNA are insufficient for providing a sufficiently large Rg to match the Rgs observed. Assuming that all of the protein components remain attached (as evident in **Fig. 5a**), the increased Rg may be explained by an extension of histone components away from the center of mass. This requires a disruption of the octameric histone and may be characterized by H2A-H2B dimers bound but positioned somewhere else along the DNA. (**b**) Comparison of Kratky profiles for the 601-NCP 200 ms kinetic intermediate in no sucrose (red circles) and the NCP model shown in the inset (black curve). The lack of a sharp peak in the data at q ≈ 0.045 Å^{-1} indicates a structure without a compact globular core. The disagreement at high q is likely a result of scattering from the flexible disordered histone tails.

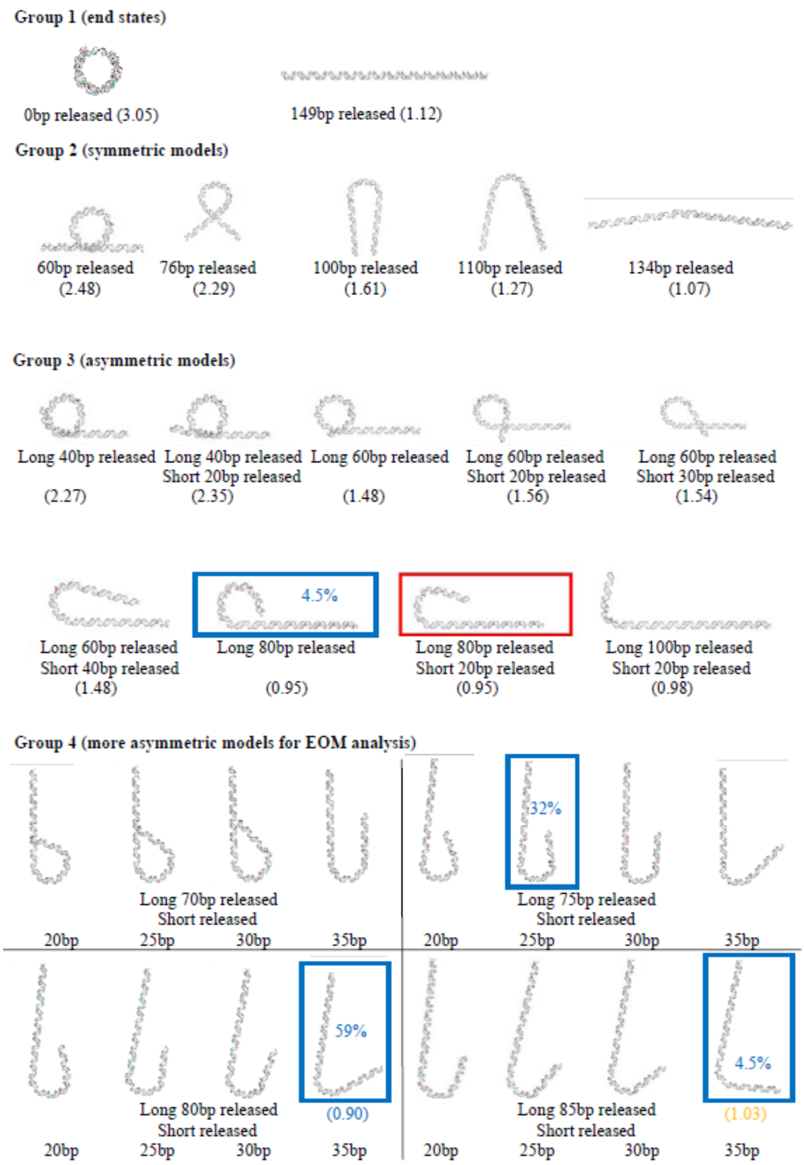

Supplementary Figure 8 Chi square and EOM analysis of models to determine the conformation of the 200 ms 601-NCP kinetic intermediate and first 160 ms 5S-NCP. These symmetric and asymmetric DNA models were generated using Discovery Studio (Accelrys) and Nucleic Acid Builder. The curved portions of these models were migrated from the DNA component of crystal structure (pdb: 1AOI). $\overline{\chi^2}$ was calculated using Supplementary Eq. 5. The calculated $\overline{\chi^2}$s between the experimental SAXS profiles and the SAXS profiles calculated from the models are reported in parentheses below each model. Black $\overline{\chi^2}$s are calculated from fits to single models, whereas blue and yellow $\overline{\chi^2}$s are calculated from fits to the ensemble of models selected by EOM analysis. The best fitting single model using chi square analysis ($\overline{\chi^2} = 0.95$) is the 'J' shaped asymmetric DNA (Long 80bp released, Short 20bp released) in the red box. The best fitting ensemble of models using chi square analysis ($\overline{\chi^2} = 0.90$) is highlighted by blue boxes. The population of each model for the ensemble is reported as a percentage inside each box. The improvement in $\overline{\chi^2}$ value for the ensemble of models (0.90) compared to the single models (0.95) reveals better agreement with experimental data. Applying chi square analysis to the first 160 ms data for the 5S-NCP using single models and an ensemble of models selects the asymmetric DNA model (Long 85bp released, Short 35bp released) with $\overline{\chi^2} = 1.03$. This suggests that the 5S-DNA may also unwrap asymmetrically.
